# Cost-effectiveness of a community-based dementia support model: evidence from a real-world economic evaluation

**DOI:** 10.1186/s12962-026-00782-5

**Published:** 2026-06-16

**Authors:** Rachel L. King, Stuart Warren, Elizabeth Vass, Benjamin T. Sharpe, Kian Beaumont, Stewart Seymour, Samuel Bell, Rosana Pacella, Antonina Pereira

**Affiliations:** 1 Institute of Psychology, Business and Human Sciences, University of Chichester, Bishop Otter Campus, College Lane, Chichester, West Sussex PO19 6PE UK; 2 Institute for Lifecourse Development, University of Greenwich, Old Royal Naval College, Park Row, London, SE10 9LS UK

**Keywords:** Supportive care, Multicomponent, Dementia, Costs, Wellbeing, Quality of life, Care partners, Cost-effectiveness, Sage House Model

## Abstract

**Background:**

The prevalence of dementia is currently set to rise to 1.7 million by 2040, with associated costs estimated at £90 billion. Given its substantial impact on quality of life (QoL) and the growing societal and financial burden, identifying efficient approaches to dementia support is a key policy priority. Current policy direction emphasises a shift toward community-based models of care; however, economic evidence is required to determine whether such approaches can improve outcomes while representing cost-effective use of limited healthcare resources. This study presents a cost-effectiveness analysis of the Sage House Model, a community-based dementia support intervention integrating NHS diagnostic services, third-sector provision, and local partnerships within a single hub.

**Methods:**

A preliminary cost-effectiveness analysis was conducted using a natural experimental design comparing individuals with dementia accessing the Sage House Model (*n* = 65) to those receiving usual care (*n* = 153). Health-Related Quality of Life (HRQoL) was assessed using a single measure, alongside health and social care utilisation, over a three-month period. Resource use was valued from a health and social care perspective. Incremental costs and outcomes were estimated to assess cost-effectiveness.

**Results:**

The Sage House Model was associated with lower incremental costs and higher incremental QALYs compared to usual care over the three-month time horizon and was likely to be cost-effective, with a 72.2% probability at a £20,000 willingness-to-pay threshold and 74.7% at £30,000 per QALY.

**Conclusions:**

These findings provide preliminary evidence that community-based dementia support approaches, such as the Sage House Model, may represent a promising strategy for improving outcomes alongside more efficient use of health and social care resources, and therefore warrants larger scale investigation.

**Supplementary Information:**

The online version contains supplementary material available at https://doi.org/10.1186/s12962-026-00782-5.

In the UK, an estimated 982,000 people are currently living with dementia, a figure projected to rise to between 1.4 and 1.7 million by 2040 [1, 2]. The increasing prevalence presents significant societal and economic challenges, with associated costs expected to rise to £90 billion [2–4]. Dementia affects both cognition and quality of life [5, 6], and significantly compromises an individual’s ability to live independently [7–11]. Beyond its impact on individuals, dementia increases demand for health care services, resulting in a substantial economic burden [2, 12]. In response to this growing burden, policy has increasingly emphasised a shift toward community-based models of care; however, there remains a lack of robust real-world economic evidence to determine whether these approaches can improve outcomes while ensuring efficient allocation of constrained health and social care resources.

For over a decade, improving support for people with dementia has been a key societal objective [13–18]. However, despite this sustained focus, existing models of care often remain fragmented and resource-intensive, highlighting the need for approaches that can deliver coordinated support while remaining economically sustainable. In this context, there is increasing interest in integrated community-based models of care that aim to improve outcomes while making more efficient use of health and social care resources.

One example of an integrated community-based approach is the Sage House Model, developed by the charity Dementia Support and implemented since 2018 in Tangmere, UK: https://www.dementiasupport.org.uk/sage-house. The model is designed as a comprehensive “one-stop” hub that brings together NHS diagnostic services, psychosocial interventions, and practical support into a single dementia-friendly community-based setting. By integrating multiple components of care, including diagnostic assessment, personalised support, and coordinated access to wider community services, the model aims to address fragmentation in existing provision, improve access to support, and reduce reliance on more resource-intensive health and social care services. The Sage House Model is developed using a co-design approach that enables adaptation to local community needs while maintaining its core components, supporting potential scalability and wider implementation across settings. The present study evaluates the impact of the Sage House Model on health-related quality of life (HRQoL) and assesses its cost-effectiveness relative to usual care, providing real-world evidence to inform resource allocation. 

The scope of the Sage House Model can be considered in relation to established frameworks of dementia support [19, 20], which emphasise the importance of integrated, multi-domain care. The model spans key domains including identification and management of needs, psychological wellbeing, practical support, and care coordination. This aligns with recent policy direction, including the Department of Health and Social Care (2025) 10-year plan, which highlights the need to shift away from hospital-centric models toward more integrated, community-based care [21]. By bringing together NHS, third-sector, and community services, the Sage House Model has the potential to improve access to support while supporting more effective use of health and social care resources.

In addition to the scope of the model, it is important to consider whether it delivers meaningful wellbeing outcomes for people with dementia and care partners. Preliminary evidence suggests that individuals accessing the Sage House Model experience higher quality of life, wellbeing, and life satisfaction [22], alongside improvements in care partner outcomes. These findings are consistent with previous research demonstrating that multicomponent supportive care interventions can improve wellbeing outcomes in both formal and community-based settings [23–28]. Importantly, such approaches may also generate efficiencies within health and social care systems, for example through reduced service utilisation or delayed institutionalisation [28]. However, it remains unclear whether the wellbeing benefits associated with access to the Sage House Model translate into improvements in HRQoL and whether the model represents a cost-effective use of health and social care resources.

Dementia is a progressive neurodegenerative condition requiring long-term care, with a median survival time estimated at approximately 5 years [29]. Given the chronic nature of the condition, short-term approaches to supportive care are often insufficient, highlighting the need for sustained and coordinated support across the disease trajectory [30–34]. The Sage House Model is designed to provide continuous support from pre-diagnosis through to end-of-life, enabling continuity of care across this trajectory. Over this time horizon, costs span multiple sectors, including health care, social care, and informal care, alongside wider economic impacts [2]. Given this complexity, integrated models, such as the Sage House Model, may generate both direct and indirect cost savings, for example through reduced service utilisation, and broader economic benefits, such as supporting care partners to remain in employment. It is therefore important to evaluate the model, not only in terms of effectiveness, but also in terms of its cost-effectiveness and impact on health and social care resources.

Previous work has shown that individuals with access to the Sage House Model demonstrate higher quality of life, life satisfaction, and wellbeing compared to usual care [22]. Building on these findings, the present analysis extends this to examine HRQoL and patterns of engagement with health and social care services. The aim of this study was to conduct an initial economic evaluation of the Sage House Model to provide preliminary estimates of cost-effectiveness and generate feasibility-related information to support future larger-scale research. In doing so, the study provides early evidence regarding the potential of an integrated, community-based model of dementia care to improve outcomes while supporting efficient use of health and social care resources.

## Method

### Ethical approval

This study was approved by the University of Chichester Research Ethics Committee (2223_05). All procedures were conducted in accordance with the Declaration of Helsinki, and participants provided informed consent prior to participation using an approach appropriate for individuals living with dementia [35].

### Service and stakeholder engagement

Service and stakeholder engagement informed study design and conduct. Meetings with Sage House representatives, including individuals with lived experience as family carers and providers of dementia support, contributed to refinement of study aims and outcome selection.

### Study design

This study employed a cross-sectional, between-subjects natural experimental design [36] to retrospectively assess differences in health and social care engagement over a three-month period, alongside current HRQoL, between participants with access to Sage House and a usual care comparator group. A three-month recall period was selected as a pragmatic balance between capturing recent variation in health and social care resource use while minimising potential recall bias associated with memory decay and telescoping errors [37, 38]. By leveraging real-world variation in service access, this approach aimed to evaluate the association between Sage House engagement and HRQoL outcomes. Given the non-randomised design, there is potential for confounding between groups; therefore, analyses were adjusted for baseline differences in functional independence (FAI), and results are interpreted as associative rather than causal. This economic evaluation was conducted in accordance with established methodological and reporting standards for observational comparative effectiveness research (GRACE) [39–41], and health economic evaluation (CHEERS) [42, 43]. Please see section  5 of the supporting materials for further detail.

### Sage House intervention

The Sage House intervention is a third-sector-led model delivered within a community-based setting. Participants in the intervention group had access to usual care (healthcare and local support services) in addition to access to the Sage House Centre, located in Tangmere, UK.

The model comprises several core components, including on-site diagnostic assessment, allocation of a dedicated support worker (Wayfinder), and provision of respite day care (Daybreaks). Participants also had access to a range of optional psychosocial interventions, including cognitive stimulation, physical activity, social support, and educational programmes, enabling personalised engagement aligned with individual needs.

**Fig. 1:**
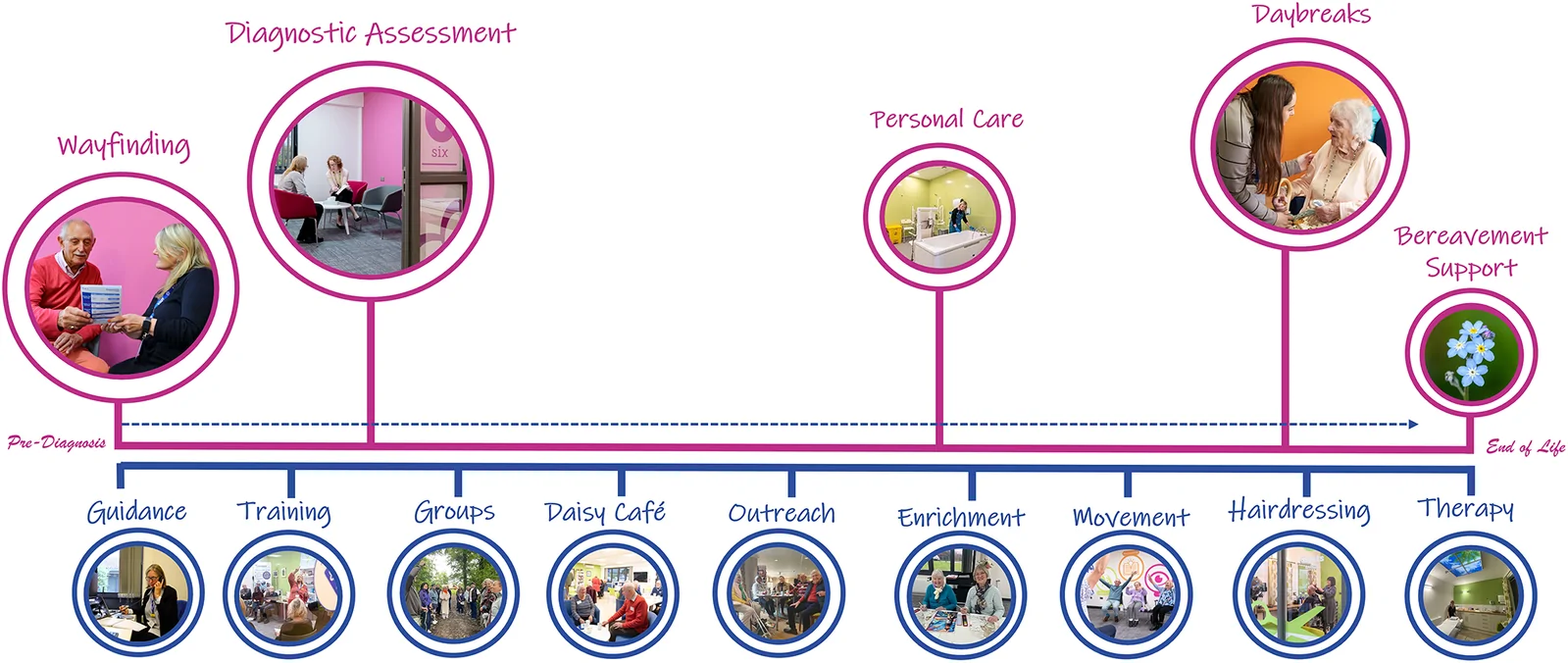
Schematic overview of the sage house model, illustrating core components and service pathways across the dementia care trajectory

Practical support is embedded within the model through coordinated access to additional services, including facilitated referrals to local services, legal and advisory support, and personal care provision. The model integrates multiple components of dementia support within a single setting to facilitate coordinated service delivery. An overview of the key components of the intervention are presented in Fig. 1, with further details provided in Table S1 in the Supporting Materials.

### Model engagement

Engagement with the Sage House intervention varied across participants. The participants in the intervention group had been attending Sage House for an average of 19.54 months (*SD* = 20.4, *Minimum* = 4, *Maximum* = 108) and over the three-month period there was an average of 37.68 (*SD* = 35.88, range: 5-209) contacts per person, indicating substantial variation in intensity of engagement across participants. Please note that one participant attended activities that were run at the centre prior to formal opening.

### Usual care comparator

The usual care comparator was recruited through the Join Dementia Research (JDR) Network and comprised individuals receiving ongoing healthcare and any supportive services available within their local community, without access to Sage House. The use of a broad usual care comparator reflects routine practice [36, 44] and is consistent with recommendations for pragmatic evaluations of community-based interventions [45], ensuring that cost-effectiveness estimates are relevant to real-world decision contexts [46].

### Setting and sample

#### Setting

The study was conducted in a community-based setting involving individuals living with dementia and their care partners accessing routine health and social care services across the south of England, primarily within the southeast region.

#### Recruitment

Participants living with dementia and their care partners were recruited through multiple channels, including study advertisements, word of mouth, and the Join Dementia Research network. Recruitment at Sage House was supported by staff who provided study information and facilitated contact with the research team. Data collection occurred alongside a related wellbeing evaluation [22], with additional measures included to capture engagement with health and social care services. Data were collected via self-report from individuals living with dementia and proxy-report from care partners to maximise sample inclusion. The measures used have been validated for both self- and proxy-report, with good agreement between reporting methods [47, 48].

#### Inclusion and exclusion criteria

Inclusion criteria were broad to reflect the range of individuals supported by the intervention. Care partners were required to be actively providing care for a person with dementia to ensure the sample reflected current practice. Participants in the Sage House group were required to have attended the centre within the preceding three months to ensure that cost data reflected active engagement with the service. People living with dementia were included if they had capacity to provide informed consent in accordance with the Mental Capacity Act [49]. Participants receiving full-time residential care or 24-hour in-home care were excluded, as these cases were associated with substantially higher health and social care utilisation and had the potential to disproportionately influence cost estimates. Full details of the data cleaning and selection process are provided in Figure S1 in section  2 of the Supporting Materials.

#### Sample

Table 1 presents the characteristics of the self- and proxy-report samples prior to combining dyads for analysis. Where data were available from both the person living with dementia and their care partner, health and social care utilisation data were derived from care partner reports, while HRQoL data were obtained from the individual with dementia. Following combination of self- and proxy-report data, the final sample comprised 218 participants: 153 in the usual care group and 65 with access to Sage House. Of these, 108 (49.54%) were self-reports from people living with dementia, 92 (42.20%) were proxy reports from care partners, and 18 (8.26%) were dyadic responses.

**Table 1:** Sample characteristics

Participant Type	Measure	Usual Care Mean (SD)	Sage House Mean (SD)	Comparison*
Sample of People Living with Dementia *N* = 126 (108 + 18 dyads)	*N*	89	37	
	Age	72.24 (8.12) 51–86 years	79.83 (6.44) 63–92 years	< 0.001
	Self-Report FAI	28.98 (8.14)	22.29 (9.69)	0.001
	MoCa (*n* = 118)	12.16 (5.31)	7.39 (4.58)	0.014
	Gender	Female: 37 (42%) Male: 50 (56%) Non-Binary: 2 (2%)	Female: 17 (46%) Male: 20 (54%)	0.92
	Education	≤ Secondary: 15 (17%) Post Secondary: 19 (21%) Degree: 25 (28%) Above Degree: 26 (29%) Missing: 4 (5%)	Secondary or below: 9 (24%) Post Secondary: 16 (43%) Degree: 9 (24%) Above Degree: 2 (8%)	0.01
	Ethnicity	White: 87 (98%) Asian: 2 (2%)	White: 36 (97%) Missing: 1 (3%)	1.0
	Diagnosis	AD: 31 (35%) VD: 8 (9%) EOAD: 7 (8%) FTD: 3 (3%) LBD: 6 (7%) Mixed: 8 (9%) MCI: 16 (18%) Other: 1 (1%) Dementia: 9 (10%)	AD: 17 (46%) VD: 2 (5%) Mixed: 1 (3%) Other: 2 (5%) Dementia: 14 (37%)	
Proxy Sample of Care Partners (CP) *N* = 110 (92 + 18 dyads)	*N*	70	40	
	CP Age	65.57 (9.84) 36–85 years	69.65 (9.48) 45–87 years	0.04
	Proxy-PwD FAI	16.71 (9.30)	14.10 (7.73)	0.15
	CP Gender	Female 57 (81%) Male 13 (19%)	Female 27 (67.5%) Male 13 (32.5%)	0.11
	CP Education	≤ Secondary: 7 (10%) Post Secondary: 18 (26%) Degree: 25 (36%) Above Degree: 16 (23%) Missing: 4 (5%)	Secondary or below: 9 (22.5%) Post Secondary: 17 (42.5%) Degree: 8 (20%) Above Degree: 5 (12.5%) Missing: 1 (2.5%)	0.06
	Ethnicity	White: 65 (93%) Asian: 2 (3%) Black: 1 (1%) Declined: 2 (3%)	White: 40 (100%)	0.70

* Group comparisons made using the Kruskal Wallis test or Fishers Exact Test (p values are uncorrected), FAI = Frenchay Activity Index

* Please note that diagnosis was available on the JDR system, whereas only self-report was available for the Sage House Group

* MoCa (Montreal Cognitive Assessment) data was missing for 8 participants, primarily due to time constraints reducing the sample to 118

Abbreviations: AD = Alzheimer’s Disease, VD = Vascular Dementia, EOAD = Early Onset Alzheimer’s Disease, FTD = Frontotemporal Dementia, LBD = Lewy Body Dementia, MCI = Mild Cognitive Impairment. Dementia = Unspecified Dementia

#### Group comparison of functional independence

Functional independence was assessed to evaluate baseline comparability between groups in the full sample. There was a statistically significant difference between groups, with the Sage House group scoring lower (*M* = 17.39, *SD* = 9.52) than the usual care group (*M* = 23.40, *SD* = 10.68), χ²(1) = 14.68, *p* <.001, η² = 0.06. This indicated that the group accessing Sage House were more functionally impaired than the usual care group. Functional independence was therefore included as a covariate in the subsequent analyses.

### Study protocol

Data collection was conducted either in person or remotely (via Microsoft Teams), according to participant preference. Following provision of study information and informed consent, measures were administered using a structured protocol via Qualtrics to ensure consistency across data collection modes. A member of the research team facilitated each session, guiding participants through the measures and recording responses. The protocol included collection of demographic information, health and social care utilisation, and HRQoL, alongside additional wellbeing measures reported elsewhere [22]. Sessions typically lasted approximately one hour, with breaks provided as needed to accommodate participant needs.

### Measures

#### Health service utilisation questionnaire

Health and social care utilisation were assessed using a bespoke questionnaire designed to capture service use over a three-month period (see Supporting Materials, section  3). The questionnaire recorded the frequency of contacts across a range of services, including general practitioner visits, hospital attendances, home visits by healthcare professionals, memory clinics, mental health services, respite day care, and home-based care. An open-ended response option was included to capture additional services and ensure comprehensive measurement of resource use. In line with the NHS and adult social care perspective adopted in this analysis, patient and carer out-of-pocket costs (e.g., travel or parking) and productivity losses were not included.

#### Costing information

Analyses were conducted from an NHS and adult social care perspective, with intervention delivery costs additionally considered from the provider perspective. A bottom-up costing approach was used to estimate individual-level resource use associated with access to the Sage House Model. Resource use was measured over a three-month period and valued in 2022 GBP using national unit cost sources, including the National Schedule of NHS Costs 2021/2022 [50] and the Unit Costs of Health and Social Care 2022 [51]. Intervention costs were estimated using provider-supplied data and allocated at the individual level to reflect the cost of Sage House service provision over the study period. Full costing details are provided in Table S2 in section  4 of the Supporting Materials.

#### Health Related Quality of Life (HRQoL)

HRQoL was assessed using the EQ-5D-3L, a widely used preference-based measure comprising five domains: mobility, self-care, usual activities, pain/discomfort, and anxiety/depression [52–54]. Health states were valued using the UK time trade-off tariff [55], generating utility scores anchored at 1 (perfect health) and 0 (death).

The EQ-5D-3L has been validated for use in people with dementia [56, 57], and proxy reporting by care partners is considered appropriate in this population [47]. Where both self- and proxy-reported data were available, self-reported HRQoL was prioritised. Quality-adjusted life years (QALYs) were estimated by multiplying the observed EQ-5D utility score by the three-month analytic horizon (0.25 years), assuming that utility remained constant over this period. As differences may exist between self-reported and proxy-reported QoL measures [58], results for both the combined and separate cohorts are presented.

The three-month time horizon reflects the period over which costs were investigated, avoiding the need for extrapolation beyond available data. While shorter than typical economic evaluations, this approach is appropriate for exploratory, real-world analyses and is supported by methodological guidance where longer-term data are unavailable [42, 43, 45, 59]. This provides decision-relevant evidence while minimising reliance on assumptions. Costs and outcomes were not discounted due to the short time horizon.

### Frenchay Activities Index

Functional independence was assessed using Frenchay Activities Index (FAI; [60]), a validated measure of instrumental activities of daily living. Scores range from 0 to 45, with higher scores indicating greater functional capacity. The scale demonstrates good internal consistency in the current sample (α = 0.87, 95% CI [0.85, 0.89]) and has established validity in elderly and stroke populations [60]. Previous research also supports strong self-proxy agreement [48].

### Data handling

All data handling and analyses were performed in R Version 4.3.1 [61] and utilised packages such as dampack [62]. Statistical assumptions underlying the analyses were assessed, including normality and homogeneity of variance where appropriate. Outliers exceeding ± 3.29 standard deviations were examined and excluded where necessary, with sensitivity checks conducted and reported to ensure that their removal did not materially affect results. Multiple comparisons were controlled using the false discovery rate [63].

### Analysis

A cost consequence analysis was first conducted to summarise costs and HRQoL outcomes [45, 64], with costs disaggregated by NHS and social care components. Group differences were examined using one-way ANCOVA adjusting for functional independence in the full and care partner samples. Additional covariates of age, years in education and the MoCa total score were incorporated in the PLWD only analysis. Given the semi-continuous and skewed distribution of cost data, including zero values, additional sensitivity analyses were undertaken using a Tweedie generalized linear model with log link and reported alongside the main analysis.

The primary economic evaluation estimated the incremental cost-effectiveness of the Sage House Model compared to usual care. Incremental cost-effectiveness ratios (ICERs) were calculated as the difference in mean costs divided by the difference in mean quality-adjusted life years (QALYs). ICERs were assessed against UK willingness-to-pay thresholds of £20,000–£30,000 per QALY gained [46]. Results were also expressed in terms of Net Monetary Benefit (NMB) calculated as: NMB = (ΔQALYs × λ) – ΔCosts, where λ represents the willingness-to-pay threshold. Positive NMB indicates that a strategy is likely to be cost-effective.

### Probabilistic sensitivity analysis

Uncertainty in costs and QALYs was assessed using non-parametric bootstrapping of individual-level data. Participants were resampled with replacement within each study group, preserving original group sizes. Incremental costs and QALYs were recalculated across 10,000 bootstrap replications and used to generate a cost-effectiveness plane and cost-effectiveness acceptability curve. The analysis reflects uncertainty over the three-month time horizon used for cost and QALY estimation, consistent with the observed data and avoiding extrapolation beyond the available follow-up period.

## Results

### Cost consequence analysis

Average health and social care costs and service utilisation are presented in Table 2, with and without Sage House intervention costs. Group differences in cost were assessed using One-Way ANCOVA, adjusted for differences in functional independence (FAI). Analyses were conducted both excluding and including Sage House intervention costs.

**Table 2:** Contacts and costs across NHS and social care services by group (mean costs with 95% CI)

Cost	Usual Care Control (*N* = 153) FAI = 23.40 (10.68)			Sage House (*N* = 65) FAI = 17.39 (9.52)			
	*n* (%)	Contacts (per person)	Cost Mean (CI)	*n* (%)	Contacts (per person)	Cost	Cost Savings
Health & Social Care Total Cost	134 (88%)	4305 (28.14)	£1320 £921 - £1720	57 (88%)	1334 (20.52)	£948 £661 - £1236	-£372
Health Care (NHS)	134 (88%)	913 (5.97)	£779 £516 - £1042	56 (86%)	304 (4.68)	£557 £376 - £739	-£222
Adult Social Care	39 (26%)	3392 (22.17)	£541 £248 - £835	23 (35%)	1030 (15.85)	£ 391 £197 - £585	-£150
Total Costs Including Sage House	134 (88%)	4305 (28.14)	£1320 £921 - £1720	65 (100%)	2449 (37.68)	£1271 £982 - £1560	-£49
Sage House	-	-	-	65 (100%)	1115 (17.15)	£323 £258 - £388	

Note: Raw data presented, differences in FAI have not been accounted for. Abbreviations: FAI, Frenchay Activities Index. Percentages indicate participants with service use during the study period; participants without contact were assigned zero contact and did not represent missing data

**Fig. 2:**
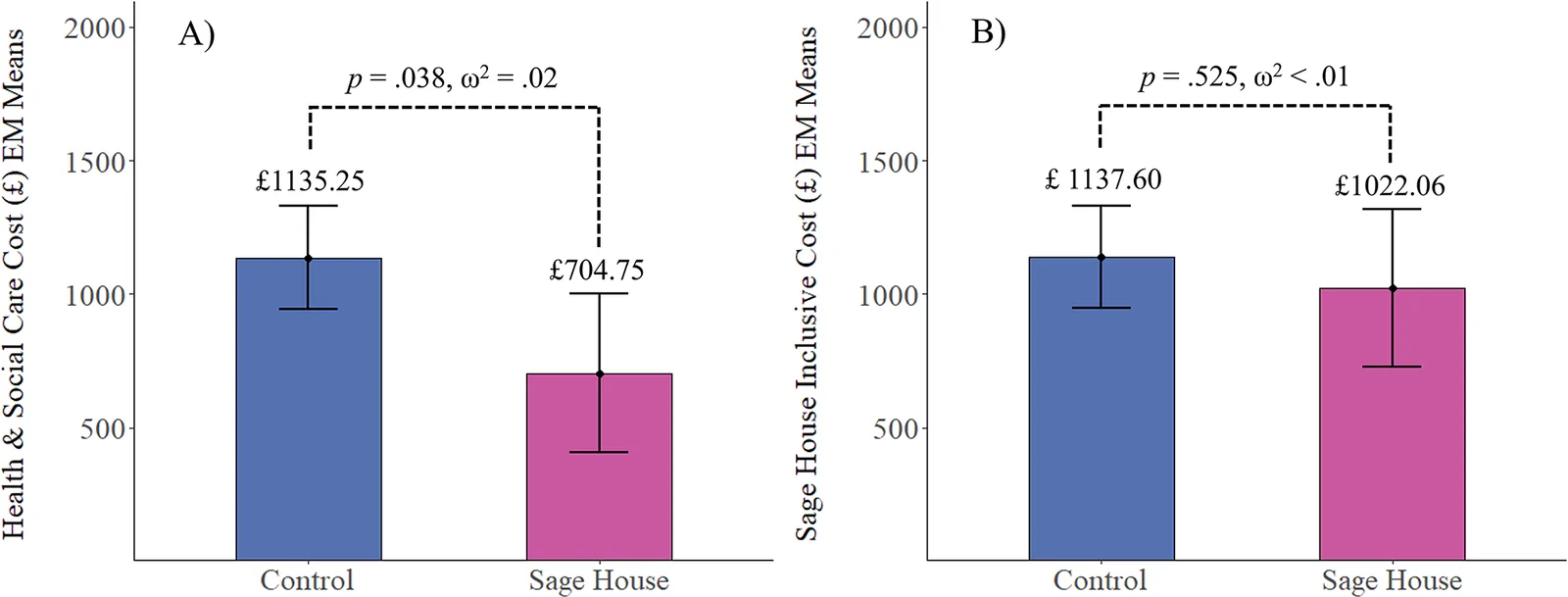
Health and social care costs split by group. **A**) Health and social care cost: Estimated marginal means and 95% CI after correcting for FAI. **B**) Costs incorporating engagement with Sage House: Estimated marginal means and 95% CI after correcting for FAI. FDR corrected p-value displayed. Note: Sage House inclusive costs include health and social care access with the cost of accessing Sage House also incorporated into the analysis

As shown in Fig. 2A, after controlling for functional independence the group with access to Sage House demonstrated significantly lower total costs to the NHS and ASC (*M* = £704.75, 95% CI [£407.92, £1001.58]) compared to the group without access to Sage House (*M* = £1135.25, 95% CI [£942.62, £1327.87]), *F*(1, 212) = 5.58, *p*_fdr_ = 0.038, ω^2^ = 0.02. On average the Sage House group incurred costs that were £430.50 lower over a three-month period, representing a modest but statistically significant difference.

Sensitivity analyses demonstrated that the direction of findings was maintained in the sample including the three outliers in the usual care group, *F*(1, 215) = 6.24, *p* =.013, ω² = 0.02. Adjusted mean costs remained lower in the Sage House group, with an estimated mean difference of £793.33. Findings were further corroborated using a Tweedie generalized linear model with log link to account for the semi-continuous and positively skewed distribution of cost data. After adjustment for FAI score, participation in the Sage House group was associated with lower expected costs (β = −0.61, SE = 0.25, *p* =.017). The exponentiated coefficient indicated that expected costs in the Sage House group were approximately 45% lower than those observed in the usual care group (cost ratio = 0.55). The estimated Tweedie index parameter (1.66) further supported the suitability of the model for the observed cost distribution.

As shown in Fig. 2B, after controlling for functional independence the group with access to Sage House was observed to have similar total costs (*M* = £1022.06, 95% CI [£726.42, £1317.68]) compared to the group without access to Sage House (*M* = £ 1137.60, 95% CI [£945.75, £1329.44]), *F*(1, 212) = 0.41, *p*_fdr_ = 0.525, ω^2^ < 0.01. On average the Sage House group incurred costs that were £115.54 lower over the three-month period, however, this comparison did not reach statistical significance.

Sensitivity analyses demonstrated that the direction of findings was maintained in the sample including the three outliers in the usual care group, *F*(1, 215) = 2.27, *p* =.133, ω² < 0.01. Adjusted mean costs remained lower in the Sage House group, with an estimated mean difference of £478.10. Findings were further explored using a Tweedie generalized linear model with log link to account for the semi-continuous and positively skewed distribution of cost data. After adjustment for FAI score, participation in the Sage House group was associated with lower expected costs; however, this difference was not statistically significant (β = −0.27, SE = 0.24, *p* =.251). The exponentiated coefficient indicated that expected costs in the Sage House group were approximately 24% lower than those observed in the usual care group (cost ratio = 0.76). The estimated Tweedie index parameter (1.66) supported the suitability of the model for the observed cost distribution.

### Comparison of HRQoL

Average impact on outcome measures is outlined in Table 3 which displays the raw data across the EQ-5D-3L measures.

**Table 3:** Outcome measures by group (mean outcome measure with 95% CI provided)

Measure	Usual Care	Sage House	Difference
	Mean 95% CI	Mean 95% CI	
EQ-5D-3L Full Health Status*	*N* = 18 (12%)	*N* = 11 (17%)	7 (5%)
EQ-5D-3L Health Index	0.58 0.53–0.63	0.61 0.54–0.68	0.03
EQ-5D-3L Visual Analogue Scale	61.77 58.22–65.33	65.86 60.76–70.97	4.09
QALYs (TH = 0.25)	0.145 0.132–0.157	0.153 0.135–0.171	0.0085

NOTE: Higher scores = Better Health Related QoL. TH = Time Horizon over three months 0.25 years

* Frequency and percentage of participants with full health status

Group differences in HRQoL outcomes were examined using ANCOVA, adjusting for baseline functional independence (FAI).

**Fig. 3:**
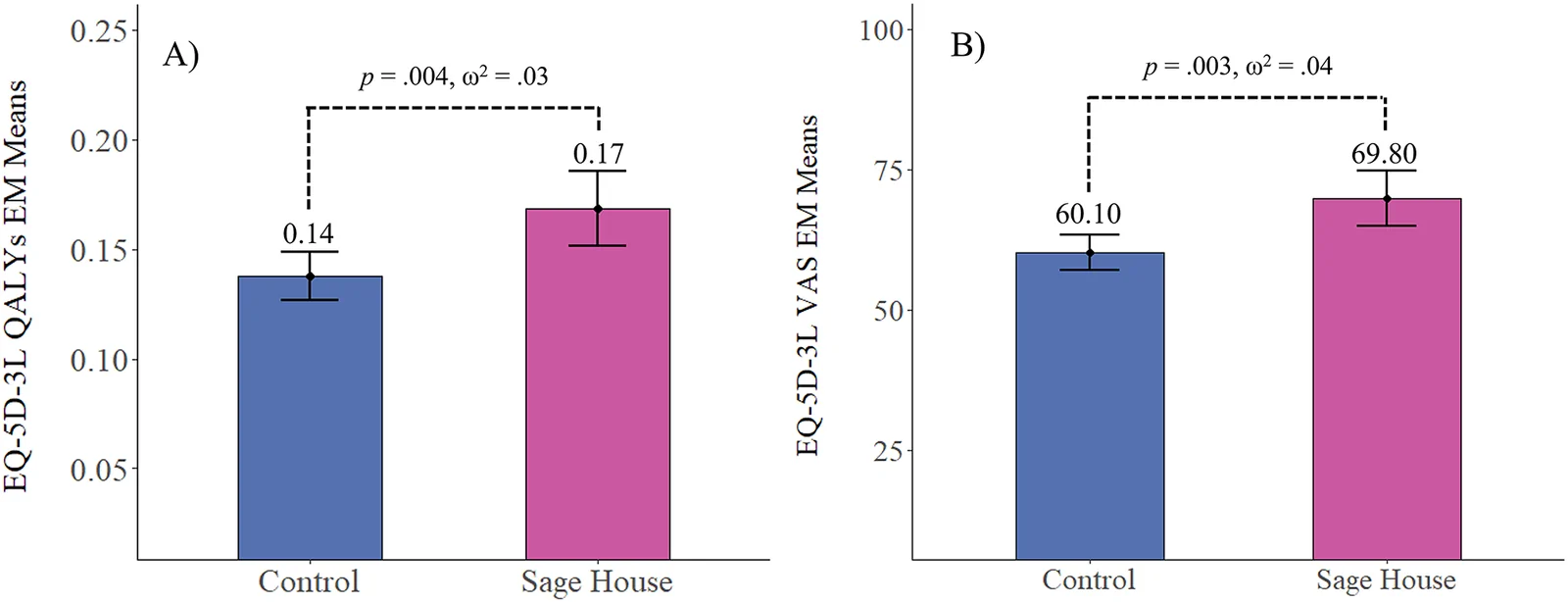
Health related Quality of Life split by group. (**A**) EQ-5D-3L QALYs (3-month time horizon): Estimated marginal means and 95% CI after correcting for FAI. (**B**) EQ-5D-3L Visual Analogue Scale: Estimated marginal means and 95% CI after correcting for FAI. FDR corrected p-value displayed

#### Quality adjusted life years (3-month time horizon)

As can be seen in the Fig. 3A, after controlling for the impact of functional independence the group with access to Sage House was observed to have a significantly higher QALYs (*M* = 0.17 [95% CI (0.15, 0.19)]) as compared to the usual care comparator (*M* = 0.14 95% CI [0.13, 0.15]), *F*(1, 215) = 8.61, *p*_fdr_ = 0.004, ω^2^ = 0.03. On average the Sage House group was scoring 0.03 QALYs higher than the comparator group, a small but significant effect.

#### Visual analogue scale

As can be seen in the Fig. 3B, after controlling for the impact of functional independence the group with access to Sage House was observed to have a significantly higher VAS scores (*M* = 69.80, 95% CI [64.90, 74.70]) as compared to the group without access to Sage House (*M* = 60.10, 95% CI [56.95–63.25]), *F*(1, 215) = 10.49, *p*_fdr_ = 0.003, ω^2^ = 0.04. On average the Sage House group was scoring 9.7 points higher than the comparator group, a small but significant effect.

### Cost effectiveness analysis

Cost-effectiveness was assessed by comparing incremental costs and QALYs between groups in the full sample (includes Sage House costs), a care partner proxy sub-sample (*n* = 110), and a PLWD sub-sample (*n* = 118) adjusted for additional covariates including age, education, and MoCA performance. Analyses were conducted both with and without influential outliers (> 3.29 SD from the group mean) and are presented in Table 4.

As can be seen in Table 4, on average the Sage House group cost less and had higher QALYs compared to the usual care comparator group. This was replicated in sensitivity analysis in the care partner sample but had mixed results in the PLWD sample where the sample was on average older and more impaired in the Sage House group.

The findings suggest that the Sage House Model is likely to be cost-effective compared with usual care. On average, the 3 month, per-patient cost of services provided via Sage House was £49.30 lower than for services provided as part of usual care (Table 4) in the full uncorrected sample. The mean Net Monetary Benefit, calculated using a willingness-to-pay threshold of £20,000 per QALY gained, was positive across the majority of analyses. Positive net monetary benefit values across analyses indicate that the intervention is likely to be cost-effective at conventional willingness-to-pay thresholds. Net health benefit values were also positive across analyses all but one analysis, reinforcing the conclusion that the Sage House model provides a net gain in health outcomes relative to its costs within the accepted UK cost-effectiveness threshold.

**Table 4:** Cost effectiveness analysis comparing costs to health related QoL (per patient over 3 months). Performed on the raw uncorrected means, the means corrected for the influence of FAI and in a control sample only including care partner reports (uncorrected means)

	TH = 0.25	Sage House		Usual Care Comparator		Incremental Costs and QALYs		Cost Effectiveness Analysis		
	Analysis Type	Total Costs M 95% CI	QALY M 95% CI	Cost M 95% CI	QALYs M 95% CI	Costs	QALYs	ICER	NMB WTP = £20,000	NHB
Full Sample	Uncorrected *N* = 218	£1271 £982, £1560	0.153 0.135, 0.171	£1320 £921, £1720	0.145 0.131, 0.157	-£49	0.009	-£5,776	£220	0.011
	FAI Corrected *N* = 218	£970 £452, £1488	0.169 0.152, 0.186	£1448 £1115, £1781	0.138 0.127, 0.149	-£478	0.031	-£15,592	£1091	0.055
	FAI Corrected Cost Outliers Removed *N* = 215	£1022 £726, £1318	0.169 0.152, 0.186	£1138 £946, £1330	0.139 0.127, 0.150	-£116	0.031	-£3,763	£730	0.037
Care Parter Sample	FAI Uncorrected *N* = 110	£1407 £1024, £1790	0.144 0.121, 0.167	£2152 £1335, £2969	0.122 0.102, 0.143	-£746	0.022	-£34,061	£1184	0.059
	FAI Corrected *N* = 110	£1281 408, 2154	0.152 0.129, 0.174	£2224 £1566, £2882	0.118 0.101, 0.135	-£944	0.034	-£27,994	£1618	0.081
	FAI Corrected Cost Outliers Removed *N* = 107	£1286 £834, £1738	0.152 0.130, 0.174	£1612 £1263, £1960	0.119 0.102, 0.135	-£326	0.033	-£9,753	£994	0.050
PLWD Sample	Uncorrected *N* = 118	£1089 £599, £1580	0.184 0.156, 0.206	£656 £478, £835	0.163 0.148, 0.178	£433	0.018	£23,535	-£65	-0.003
	FAI, Age, Education and MoCa Corrected *N* = 118	£1008 £630, £1386	0.181 0.156, 0.206	£685 £474, £897	0.163 0.149, 0.177	£323	0.018	£17,804	£40	0.002
	FAI, Age, Education and MoCa Corrected Cost Outliers Removed *N* = 116	£705 £382, £1029	0.189 0.164, 0.215	£694 £520, £868	0.163 0.149, 0.176	£11	0.027	£425	£519	0.026

Abbreviations: WTP, Willingness to Pay Threshold; ICER, Incremental Cost Effectiveness Ratio (Difference in Cost / Difference in Outcome); NMB, Net Monetary Benefit (Difference in Outcome * Willingness to Pay Threshold – Difference in Cost); NHB, Net Health Benefit (Difference in Outcome - (Difference in Cost / Willingness to Pay Threshold). Please note that estimated costs include individually calculated costs for attending Sage House (see the supplementary information for more detail on costing)

### Probabilistic sensitivity analysis

**Fig. 4:**
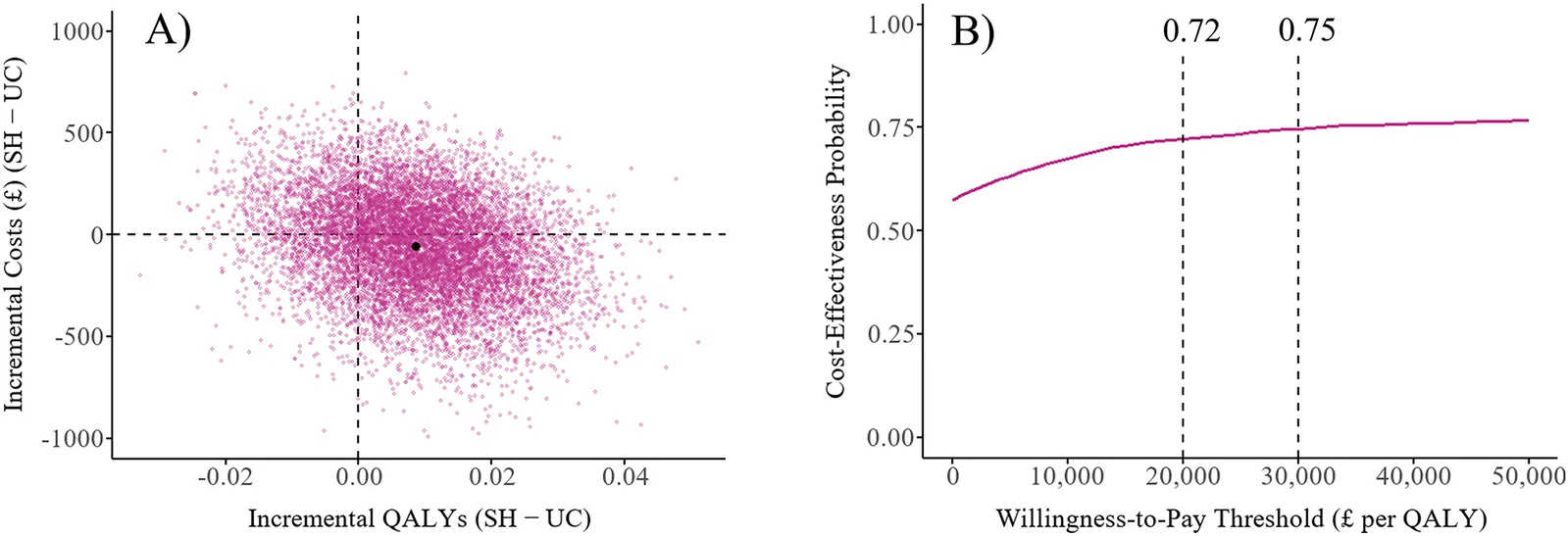
Probabilistic sensitivity analysis. **A**) Cost-effectiveness plane showing the joint distribution of incremental costs and incremental QALYs for Sage House compared with usual care, derived from 10,000 non-parametric bootstrap replications (black marker denotes the mean incremental cost–effect pair). **B**) Cost-effectiveness acceptability curve illustrating the probability that Sage House is cost-effective across a range of willingness-to-pay thresholds for a QALY. Vertical dashed lines indicate conventional NICE thresholds (£20,000 and £30,000 per QALY)

Probabilistic sensitivity analysis indicated that Sage House was cost-saving in 57.5% of simulations, with the cost-effectiveness plane (Fig. 4A) illustrating the distribution of bootstrap replications relative to the zero-cost axis. The cost-effectiveness acceptability curve (Fig. 4B) showed a 72.2% and 74.7% probability of cost-effectiveness at willingness-to-pay thresholds of £20,000 and £30,000 per QALY, respectively. These results suggest that the Sage House Model is likely to be cost-effective at conventional NICE thresholds, although uncertainty remains. Results were stable when increasing replications from 5,000 to 10,000.

## Discussion

The present analysis extends our previous findings regarding wellbeing outcomes [22], providing preliminary evidence that access to the Sage House model may be associated with lower health and social care costs alongside higher HRQoL. When the costs of delivering the intervention were incorporated, the model remained associated with broadly similar or lower overall costs and higher HRQoL compared with usual care. Sensitivity analyses using a Tweedie generalized linear model with log link to account for the skewed and semi-continuous distribution of cost data demonstrated a broadly consistent pattern of findings, supporting the robustness of the overall economic signal observed across analyses.

The probability of cost-effectiveness exceeded 70% at conventional NICE willingness-to-pay thresholds; however, the cost-effectiveness acceptability curve did not approach certainty, indicating residual decision uncertainty. This uncertainty appears to be driven primarily by variability in short-term cost estimates and relatively modest, albeit consistently positive, QALY gains. Taken together, these findings suggest that the costs of delivering the Sage House Model may be partially offset by reductions in health and social care utilisation, indicating the potential for cost-effectiveness relative to usual care.

These findings should be interpreted with caution given the observational design, short-term time horizon, and baseline differences observed between groups. Nevertheless, the consistency of the overall pattern of findings across analyses suggests that integrated, community-based dementia support models such as Sage House may have the potential to improve outcomes while supporting efficient use of health and social care resources. As such, the present study provides an important foundation for future economic evaluations and highlights the value of further investigation within larger scale investigations.

Supporting people with dementia to live well has been a priority across multiple governmental strategies, both in the UK [13–15, 65] and globally [18]. In alignment with previous work examining multicomponent supportive care approaches [28], the present findings suggests that the Sage House Model may represent a cost-effective approach to delivering integrated dementia support. While the model shares features with previous supportive care approaches, it is distinctive in providing support across the dementia journey, pre-diagnosis to end of life, and in aligning with comprehensive frameworks of care, such as that proposed by Bamford et al. (2021) [19]. Its community partnership structure also reflects current policy priorities to shift care delivery towards integrated community-based provision [21].

These findings provide preliminary evidence that such models may contribute to improving access to meaningful support whilst making efficient use of health and social care resources. However, further research is required to examine scalability, sustainability and longer-term outcomes. It is also important to interpret these findings in light of the model’s funding structure. The Sage House Model operates using a mixed funding approach rather than being fully commissioned, meaning that not all costs are borne by health and social care systems. In the present analysis, unrestricted access to the service was costed at the individual level; however, in a commissioning context, funding would more likely be allocated to defined service packages or specific components of care.

### Multicomponent supportive care approaches

The present findings align with previous evidence suggesting that multicomponent supportive care approaches may improve outcomes while maintaining or reducing costs [28]. Similar patterns have been observed in intervention such as the WHELD programme in care home settings, which integrates psychosocial interventions with staff training and has demonstrated improvements in quality of life alongside potential cost offsets [23, 24]. Comparable findings have also been reported in community and day-care settings. For example, the MAKS intervention, (which contains cognitive, functional, and social components), has been shown to maintain functional outcomes while remaining cost-neutral when broader societal costs are considered [26]. Similarly, coordinated community-based approaches, such as that evaluated by Eloniemi-Sulkava and colleagues [27] have been associated with reduced service utilisation and delayed institutionalisation. Taken together, these findings suggest that the combination of multiple supportive care components, particularly when delivered in coordinated and accessible formats, may contribute to improved outcomes without substantially increasing overall costs. The Sage House Model extends this evidence by demonstrating similar patterns within a community-based, integrated service model that spans the full dementia care pathway.

The outcomes examined in previous studies also highlight important areas for further evaluation of the Sage House Model. Although informal care was not included in the present analysis, it represents a substantial component of the overall economic burden of dementia [2–4] and should be incorporated in future research. In particular, services such as Daybreaks may have implications for care partner productivity and the ability to remain in employment. Similarly, findings from the intervention investigated by Eloniemi-Sulkava and colleagues [27], emphasise the importance of examining longer term outcomes, including delayed institutionalisation. Components of the Sage House Model, such as respite provision, access to personal care, and care partner support, may plausibly influence these outcomes. Future longitudinal work should therefore seek to incorporate informal care costs and delayed access to institutionalisation, alongside continued evaluation of cognitive and functional trajectories.

A common feature of effective supportive care approaches is the integration of services in a manner that enables personalised and adaptable care. Central to this is the presence of a single point of contact, such as a care coordinator, which supports continuity across the dementia pathway [19]. Similar principles are reflected in “one-stop-shop” models that provide coordinated care through a dedicated keyworker alongside support for both people living with dementia and care partners [66]. The Sage House Model incorporates these elements through a partnership-based approach that integrates third-sector provision, local services, and NHS input within a single setting. This structure may facilitate coordinated care delivery while distributing financial and organisational responsibility across sectors. More broadly, this type of integrated, partnership-based model may represent an important mechanism for delivering sustainable community-based dementia care, particularly in the context of constrained health and social care resources.

### Methodological considerations

In line with the GRACE principles for evaluating observational studies [39, 40], this analysis incorporates several strengths, including the use of real-world data, a clearly defined population, and consistent data collection across groups. Outcomes were assessed using a standardised HRQoL measure, resource use was captured in detail, and differences in functional independence were measured and adjusted for in the analysis. However, several limitations should be considered when interpreting these findings and their generalisability.

While the flexibility of the Sage House Model is a key strength from an implementation perspective, it also introduces variability in how participants engage with the service. This heterogeneity in service use makes it more challenging to define and evaluate the intervention consistently across individuals. From an economic perspective, variation in intensity and type of service use may influence both costs and outcomes, contributing to uncertainty in cost-effectiveness estimates. Further research is therefore needed to characterise patterns of engagement and identify the level and type of service use associated with optimal outcomes.

The present study utilised a broad usual care comparator, defined as access to non-integrated support services within participants’ local areas. While this approach reflects routine practice and is consistent with recommendations for pragmatic evaluations [36, 45, 46], it also introduces heterogeneity in the comparator condition. As a result, “usual care” cannot be precisely characterised, which may limit the specificity of comparisons between groups. Future research should seek to capture more detailed information on service use within the comparator group, enabling more clearly defined comparisons and, where possible, evaluation against specific alternative models of care.

In the present study, health economic data were collected alongside wellbeing measures, using a combination of self- and proxy-reported data to maximise sample inclusion. While this approach enabled a larger and more inclusive dataset, it will have introduced additional variability. In particular, proxy respondents provided their own demographic information rather than that of the person living with dementia, resulting in incomplete covariate data and limiting the extent of risk adjustment in the full sample economic analysis (e.g., controlling for age, education and cognitive impairment). Future studies should seek to collect more detailed and consistent demographic information to support more robust adjustment for baseline differences [41, 67]. Moreover, although proxy reporting is considered appropriate in dementia research [47, 48], and validated measures were used, the potential for reporting differences between self- and proxy responses should be acknowledged when interpreting the findings. This is particularly the case when considering self-proxy rated EQ-5D ratings where previous work has demonstrated systematic dependencies between perspectives in dementia populations [58].

The use of retrospective recall to capture health and social care utilisation represents a further limitation, particularly given the cognitive impairments associated with dementia. Although the recall period was restricted to three months to minimise recall bias, some degree of measurement error is likely. The use of care partner reports and sensitivity analyses restricted to proxy responses provided some reassurance regarding the consistency of findings; however, these results should still be interpreted with caution. Future studies using prospective data collection would help to strengthen the robustness of cost estimates.

The use of a cross-sectional, between-subjects natural experimental design represents a further limitation. While this approach enabled an ecologically valid comparison within a real-world setting, it precluded baseline measurement of HRQoL and relied on non-random allocation to groups. As a result, residual confounding cannot be ruled out, and observed differences between groups may, in part, reflect pre-existing differences rather than the effect of the intervention.

Within the usual care sample, highly educated participants were overrepresented, likely reflecting the requirement to independently identify and register with the JDR Network platform. Baseline imbalance was particularly apparent within the PLWD sample, where cognitive difficulties may have posed an additional barrier to participation. Compared with the usual care group, participants in the Sage House PLWD subsample were significantly older, less educated, and demonstrated greater impairment on both the FAI and MoCA. In contrast, the care partner proxy sample appeared more comparable across groups, with no significant differences observed in proxy-rated functional independence. This relative comparability may have reduced baseline imbalance within these analyses and contributed to the greater consistency observed between the adjusted and unadjusted economic models in the care partner subsample. Future research would benefit from the use of matched comparison groups, particularly when recruiting through platforms such as JDR, in order to improve representativeness of the sample and minimise baseline imbalance between groups.

A further limitation is the use of a three-month time horizon for QALY estimation. While this reflects the period over which costs and outcomes were measured, it limits the ability to capture longer-term changes in health outcomes and cost trajectories. As such, estimates of cost-effectiveness should be interpreted with caution. Given the progressive nature of dementia and the sustained support provided by the Sage House Model across the care pathway, longer-term evaluation is needed to more fully assess its economic impact. Longitudinal study designs would enable more accurate estimation of cumulative costs and outcomes over time.

### Future directions

Building on this preliminary analysis, larger-scale longitudinal studies are needed to extend and confirm the present findings. Following individuals with and without access to the Sage House Model over time would enable evaluation of longer-term outcomes, including changes in QALYs, time to institutionalisation, and trajectories of cognitive and functional decline. A longitudinal design would also allow for improved control of individual differences and reduce reliance on retrospective recall by enabling prospective tracking of health and social care utilisation. Since this research was undertaken, the Sage House Model has been replicated across additional sites, creating further opportunities for larger-scale investigation across multiple locations. It is also recommended that future studies adopt a broader societal perspective which would allow inclusion of outcomes such as productivity loss and informal care. Such approaches would provide a more comprehensive assessment of cost-effectiveness and support stronger evidence for decision-making in line with best-practice economic evaluation frameworks [45].

## Conclusion

Overall, this study contributes to the growing evidence base on how integrated, community-based service models can influence both patient outcomes and patterns of health and social care utilisation. The findings provide preliminary evidence that the Sage House Model may offer a promising community-based approach to improving HRQoL for people with dementia while supporting efficient use of health and social care resources. These observations are particularly relevant in the context of ongoing efforts to shift dementia care away from hospital-centric models toward more integrated, community-based provision. In this regard, the Sage House Model provides a practical example of how coordinated, partnership-based approaches may be implemented within real-world settings. Further large-scale longitudinal research is required to confirm these findings and to more fully evaluate long-term outcomes, including cost-utility, cognitive and functional trajectories, transitions to residential care, and the contribution of informal care. Taken together, these findings support the potential role of integrated, community-based dementia care models in addressing rising demand while ensuring efficient use of constrained health and social care resources.

## Supplementary Information

Below is the link to the electronic supplementary material.


Supplementary Material 1


## Data Availability

The datasets generated and/or analysed during the current study are available from the corresponding author on reasonable request.
