# Supplementary material for: Cost-effectiveness of a community-based dementia support model: evidence from a real-world economic evaluation
Source: Cost Eff Resour Alloc. 2026 Jun 16;24:111. doi: 10.1186/s12962-026-00782-5 (PMC13531959; doi:10.1186/s12962-026-00782-5)
Supplement: Supplementary file 1 [file 12962_2026_782_MOESM1_ESM.docx]

**Section 1: Alignment with Bamford Framework**

**Table S1**

*The Sage House Model within the Context of the Bamford et al (2021) Framework*

| **Bamford Theme** | **Component** | **Description of Sage House Provision** |
| --- | --- | --- |
| **Timely Identification and Management of Needs** | Diagnosis | **Indirect**: DAS conducting diagnostic assessment on site. Centre compliments by providing pre and post diagnostic support. |
|  | Review & Care Planning | **Indirect**: DAS conducts annual review on site. Wayfinder compliments with ongoing support, check-in and sign posting. |
|  | Supporting Physical Health | **Indirect**: Staff monitor changes, signpost and the centre provide access to activities to promote health (e.g., physical activities, Chiropody/Podiatry and now also Ear/Hearing care). |
|  | Contingency and future planning | **Direct**: Wayfinder meetings, onsite access to solicitors, CAB and training provided to support planning. |
|  | Carer Assessment and Review | **Indirect**: Wayfinder meetings and review. Support with referrals to carers assessment when needed. |
| **Understanding and Managing Dementia** | Maintain Cognition | **Direct**: Access to enrichment, CST, social engagement and physical activities. |
|  | Information Provision | **Direct**: Access to reception, Wayfinder service, training, solicitors, CAB and social support groups. |
|  | Strategies for Living with Dementia | **Direct**: Wayfinder service, support groups and access to training. |
|  | Managing Behavioural Symptoms | **Direct**: Wayfinder service, support groups and access to training. |
| **Psychological and Emotional Wellbeing** | Supporting Emotional Wellbeing | **Direct**: Wayfinding service, support groups and sign posting to local services with warm handover. |
|  | Peer Support | **Direct**: Support groups, outreach groups, café and enrichment activities. |
|  | Supporting Relationships | **Direct**: Activities and events to attend together in supportive environment, personal care, referrals to counselling when required and support to enable independent Wayfinding access. |
|  | Meaningful Activities | **Direct**: A range of enrichment activities (e.g., reminisce activities, arts, music), physical activities and social events. |
| **Practical**  **Support** | Maintaining Independence & Managing Risk | **Direct**: Training, access to try accessibility-based technology, personal care and Daybreaks (support to complete ADL during Daybreaks). Dementia friendly design of the centre supports independent attendance. Wayfinding service and training to support with identifying and managing risk. |
|  | Safeguarding & Advocacy | **Direct**: Support provided to make complex decisions, safeguarding policies in place and Wayfinder can act as advocate or make a referral to outside organisation when required with a warm handover approach. |
|  | Having a Break | **Direct**: Daybreaks, independent time in the café when activities are on (emotional break) and supports personal care. |
|  | Support with Finances | **Direct**: Support from Wayfinder, CAB and access to solicitor on site. Can also support referral to local authority for assessment with warm handover approach. Also tiered Daybreaks costs and free Wayfinding. |
| **Integrating Support** | Named Point of Contact | **Direct**: Wayfinder, Sage House Centre and when accessing Daybreaks senior support worker. |
|  | Care Coordination | **Indirect**: Wayfinder acts in a complimentary manner by providing warm handover to different services and acts as a consistent point of contact. |
|  | Managing Transitions | **Indirect**: Wayfinder acts in a complimentary manner by providing consistent point of contact and support during times of transition. May also help facilitate contact. |

*DAS = NHS Dementia Assessment Service, CAB = Citizens Advice Burio,*

*Warm hand over = directly facilitating hand over by supporting contact, paperwork and referrals.*

**Section 2: Data Processing Flow Chart**

**Figure S1**

*Data Selection Flow Chart*

*Note: In the PLWD sub-group analysis 8 participants were lost due to missing data. Missingness was mainly due to practical considerations (e.g., running out of time).*

**Section 3: Health History Questionnaire**

We will ask you a few questions about your engagement with different health or support services you may have engaged with in the past 3 months.

We understand that answering some of these questions might be difficult. Please be aware that if you don’t know the exact answer giving an estimate is okay. It is fine to ask someone to assist you in answering the questions.

1. **In the last 3 months have you visited the GP?**

No

Yes	Approximately how many times ____

2. **In the last 3 months have you visited the hospital?**

No

Yes	Why did you attend?

Visit to see the nurse	Approximately how many times ____

Visit to see the consultant	Approximately how many times ____

Visit to see the physiotherapist	Approximately how many times ____

Visit to see the occupational therapist	Approximately how many times ____

Visit to see the speech and language therapist	Approximately how many times ____

Visit to see the podiatrist	Approximately how many times ____

Visit to A & E	Approximately how many times ____

Was an ambulance called? Approximately how many times ____

3. **In the last 3 months have you visited the memory clinic?**

No

Yes	Why did you attend?

Assessment	Approximately how many times ____

Memory Training	Approximately how many times ____

Information	Approximately how many times ____

Other	Approximately how many times ____

If other is selected: Why did they attend? _____________

4. **In the last 3 months have you been visited at home by a health care worker?**

No

Yes	If yes please specify who visited you:

Nurse	Approximately how many times ____

Doctor	Approximately how many times ____

Paramedic	Approximately how many times ____

Occupational therapist	Approximately how many times ____

Physiotherapist	Approximately how many times ____

Speech and language therapist	Approximately how many times ____

Podiatrist	Approximately how many times ____

Other, _____________	Approximately how many times ____

5. **In the last 3 months have you been visited at home by a care staff to support daily living?**

No, I live in a residential care home.

No, I have not been visited by a care worker.

Yes

Approximately how long are the visits? _____

How many times a day do they visit? _____

How many times a week do they visit? _____

Have they been attending for the last 3 months? Yes/No

If not, how long have they been attending for? _______

6. **In the last 3 months have you accessed any mental health support services?**

No

Yes	If yes please tell us why you visited:

Crisis support	Approximately how many times ____

Assessment	Approximately how many times ____

Treatment	Approximately how many times ____

7. **In the last 3 months have you accessed respite day care (not at Sage House)?**

No

Yes	How many times did you visit? _____

8. **In the last 3 months have you accessed residential respite care?**

No

Yes	Approximately how long did you stay for? ___ days

**Pop up to appear only if they specified attending Sage House**

9. **In the last 3 months have you visited Sage House?**

No

Yes	Why did you attend?

Way Finding	Approximately how many times ____

Day breaks	Approximately how many times ____

Activities	Approximately how many times ____

Bathing	Approximately how many times ____

Hair dressing	Approximately how many times ____

Therapy sessions	Approximately how many times ____

Foot care	Approximately how many times ____

Assessment	Approximately how many times ____

Other	Approximately how many times ____

**Section 4: Costing Information (All responses refer to the person living with dementia unless otherwise specified)**

**Table S2**

*Costing Information for Accessing Health and Social Care (All unit costs are reported in 2022 GBP)*

| **Service** | **Staff Member** | **Cost** | **Retrieved from** | **Location** | **Notes** |
| --- | --- | --- | --- | --- | --- |
| **GP Cost** | **General Practitioner** | **£42.00** | [**Unit Costs of Health and Social Care Programme 2022-2027**](https://www.pssru.ac.uk/unitcostsreport/) | Table 9.4.2  Unit costs for a GP  Page 66-67 | Appointment of 9.22 minutes  Includes direct care and qualification costs. |
| **Hospital** | **Nurse** | **£75.58** | [**National Schedule of NHS Costs 21/22 V3**](https://www.england.nhs.uk/costing-in-the-nhs/national-cost-collection/) | Community Health Services (CHL) Worksheet  Row 150 (CC: N29AF) | Other Specialist Nursing Adult Face to Face |
|  | **Consultant** | **£174.64** |  | Outpatient Care (OP)  Worksheet  Row 487 (CC: WF01A) | Consultant Led  General Internal Medicine Face to Face Follow Up |
|  | **Physiotherapist** | **£100.14** |  | Outpatient Care (OP)  Worksheet  Row 1878 (CC: WF01A) | Non-Consultant Led Physiotherapy Service Non-Admitted Face to Face Follow Up |
|  | **Occupational Therapist** | **£102.25** |  | Outpatient Care (OP)  Worksheet  Row 1886 (CC:WF01A) | Non-Consultant Led Occupational Therapy Service Non-Admitted Face to Face Follow Up |
|  | **Speech and Language Therapist** | **£222.42** |  | Outpatient Care (OP)  Worksheet  Row 1894 (CC: WF01A) | Non-Consultant Led Speech and Language Therapy Service Non-Admitted Face to Face Follow Up |
|  | **Podiatrist** | **£90.33** |  | Outpatient Care (OP)  Worksheet  Row 1902 (CC: WF01A) | Non-Consultant Led Podiatrist Service Non-Admitted Face to Face Follow Up |
|  | **Accident and Emergency**  [**Type 1 Department**](https://www.england.nhs.uk/statistics/wp-content/uploads/sites/2/2019/07/AE-Attendances-Emergency-Definitions-v4.0-final-July-2019.pdf) | **£257.94** |  | Emergency Care (EC) Worksheet  Row 69 (CC: VB09Z) | Emergency Medicine Category 1 with category 1 or 2 treatment – Not Admitted |
|  | **Ambulance** | **£390.08** |  | Ambulance (AMB) Worksheet  Row 8 (CC: 3) | Ambulance: See and convey |
|  | **Other: X-Ray** | **£41.41** |  | Imaging (IMG) Worksheet  Row 54 (CC: PF) | Imaging: Outpatient  Plain Film |
|  | **Other: MRI** | **£188.11** |  | Imaging (IMG) Worksheet  Row 55 (CC: RD01A) | Imaging: Outpatient  MRI Scan Without Contrast Adult |
|  | **Other: Ultrasound** | **£77.81** |  | Imaging (IMG) Worksheet  Row 87 (CC: RD40Z) | Imaging: Outpatient  Ultrasound Scan Without Contrast < 20 minutes |
|  | **Other: Optometrist** | **£106.79** |  | Outpatient Care (OP)  Worksheet  Row 1946 (CC: WF01A) | Non-Consultant Led Optometry Service Non-Admitted Face to Face Follow Up |
|  | **Other: Audiologist** | **£76.08** |  | Audiology (AUD)  Worksheet  Row 13 (CC: AS08) | Follow-up, Adult  Face-to-Face |
|  | **Other:**  **Urology Service** | **£130.85** |  | Outpatient Care (OP)  Worksheet  Row 1146 (CC: WF01A) | Non-Consultant Led Urology Service Non-Admitted Face to Face Follow Up |
|  | **Other: Pharmacist** | **£55.00** | [**Unit Costs of Health and Social Care Programme 2022-2027**](https://www.pssru.ac.uk/unitcostsreport/) | Table 8.2.1 Annual and unit costs for community-based scientific and professional staff | Band 6 – Pharmacist  Based on community-based scientific and professional staff because home visit indicated |
| **Memory Clinic** | **Assessment** | **£838.89** | [**Unit Costs of Health and Social Care Programme 2022-2027**](https://www.pssru.ac.uk/unitcostsreport/) | Table 7.6.1 Cost per new patient associated with memory assessment service.  Page 53 | Mean assessment costs from Table 7.6.1  (minus cost of MRI to avoid duplication)  £1,027 – £188.11 |
|  | **Post-Diagnostic Support** | **£206 (£412/2)** |  | Table 7.6.1 Cost per new patient associated with memory assessment service.  Page 53 | Median cost of post diagnostic support for 6 months (£412) divided by 2 to reflect the 3-month period (£206) |
| **Mental Health** | **Initial Assessment**  **CC18 Low Need**  **Cluster based on EuroQoL Mood** | **£282.28** | [**National Schedule of NHS Costs 21/22 V3**](https://www.england.nhs.uk/costing-in-the-nhs/national-cost-collection/) | Mental Health Care Clusters (MHCC)  Worksheet  Row 46 (CC: MHCC18) | Mental Health Care Cluster Initial Assessment  Cognitive impairment (low need) |
|  | **Initial Assessment**  **CC19 Moderate Need**  **Cluster based on EuroQoL Mood** | **£292.16** |  | Mental Health Care Clusters (MHCC)  Worksheet  Row 47 (CC: MHCC19) | Mental Health Care Cluster Initial Assessment  Cognitive impairment or Dementia (moderate need) |
|  | **Initial Assessment**  **CC20 High Need**  **Cluster based on EuroQoL Mood** | **£296.75** |  | Mental Health Care Clusters (MHCC)  Worksheet  Row 48 (CC: MHCC20) | Mental Health Care Cluster Initial Assessment  Cognitive impairment or Dementia (high need) |
|  | **Telephone Appointment**  **Crisis Support** | **£12.33** |  | Table 8.2.1 | Band 4 Staff Costs  Median call length 20 minutes |
|  | **Mental Health Support**  **CC18 Low Need**  **Cluster based on EuroQoL Mood** | **£7.02** |  | Mental Health Care Clusters (MHCC)  Worksheet  Row 23 (CC: MHCC18) | Mental Health Care Cluster  Cognitive impairment (low need) |
|  | **Mental Health Support**  **CC19 Moderate Need**  **Cluster based on EuroQoL Mood** | **£13.70** |  | Mental Health Care Clusters (MHCC)  Worksheet  Row 24 (CC: MHCC19) | Mental Health Care Cluster  Cluster 19: Cognitive impairment or dementia (moderate need) |
|  | **Mental Health Support**  **CC20 High Need**  **Cluster based on EuroQoL Mood** | **£47.05** |  | Mental Health Care Clusters (MHCC)  Worksheet  Row 25 (CC: MHCC20) | Mental Health Care Cluster  Cluster 20: Cognitive impairment or dementia (high need) |
| **Home Visits by Health Care Professional** | **Paramedic**  [**Band4/5 Average**](https://www.healthcareers.nhs.uk/explore-roles/allied-health-professionals/roles-allied-health-professions/roles-allied-health-professions/paramedic) | **47.50** | [**Unit Costs of Health and Social Care Programme 2022-2027**](https://www.pssru.ac.uk/unitcostsreport/) | Table 8.2.1 Annual and unit costs for community-based scientific and professional staff (bands) | Based on information stating the paramedics start at band 5 and move to band 6 after 2 years an average of these bands has been taken. |
|  | **Doctor** | **£42** |  | Table 9.4.2  Unit costs for a GP  Page 66-67 | Includes direct care and qualification costs (9.22) |
|  | **Nurse** | **£53.74** | [**National Schedule of NHS Costs 21/22 V3**](https://www.england.nhs.uk/costing-in-the-nhs/national-cost-collection/) | Community Health Services (CHS)  Row 70 (CC: N02AF) | Nursing: Distinct Nurse, Adult Face to Face |
|  | **Occupational Therapist**  **POC assessment** | **£99.11** |  | Community Health Services (CHS)  Row 11 (CC: A06A1) | Allied Health Professional: Occupational Therapist, Adult One-to-One |
|  | **Speech and Language Therapist** | **£128.16** |  | Community Health Services (CHS)  Row 25 (CC: A13A1) | Allied Health Professional: Speech & Language Therapist, Adult One-to-One |
|  | **Physiotherapist** | **£73.14** |  | Community Health Services (CHS)  Row 15 (CC: A08A1) | Allied Health Professional: Physiotherapist, Adult One-to-One |
|  | **Podiatrist** | **£69.50** |  | Community Health Services (CHS)  Row 19 (CC: A09A) | Allied Health Professional: Podiatrist (Tier 1 - General), Adult One-to-One |
|  | **Other: Dietitian** | **£77.41** |  | Community Health Services (CHS)  Row 10 (CC: A03) | Allied Health Professional: Dietitian |
|  | **Other: Neurologist Nurse**  **DoLs assessments** | **£118.34** |  | Community Health Services (CHS)  Row 132 (CC: N22AF) | Nursing: Specialist Nursing, Parkinson's and Alzheimer’s Nursing/Liaison, Adult, Face to face |
|  | **Other: Other Specialised Nursing** | **£75.58** |  | Community Health Services (CHS)  Row 150 (CC: N29AF) | Nursing: Other Specialist Nursing, Adult, Face to face |
|  | **Other: Mental Health High Support Need** | **£47.05** |  | Mental Health Care Cluster (MHCC)  Row 25 (CC: MHCC20) | Cluster 20: Cognitive impairment or dementia (high need) |
|  | **Other: Dementia Support** | **£13.70** |  | Mental Health Care Cluster (MHCC)  Row 24 (CC: MHCC19) | Cluster 19: Cognitive impairment (moderate need) |
| **Social Care** | **Social Worker Home Visit** | **£50.00** | [**Unit Costs of Health and Social Care Programme 2022-2027**](https://www.pssru.ac.uk/unitcostsreport/) | Table 10.1.1 Social Worker (Adult Services)  Page 77 | Costs selected with qualification included.  £42 without qualification added. |
|  | **Home Care Worker** | **£23** |  | Table 10.4.1: Costs and unit estimations for a home care worker | Costs are per weekday hour |
|  | **Authority:**  **Day Care**  **(2.5 hours)** | **£78** |  | Table 1.4.1: Costs and unit estimations for local authority own-provision day care (age 65+) | Note this is elderly provision (65+) rather than dementia specific day care. |
| **Sage House**  (Sage House unit costs were derived from internal provider costings and reflect staff time, space, and overheads apportioned per contact.) | **Wayfinder** | **£24.71** | **Sage House: Unit Cost Calculations** | Table 2 Wayfinding Costing  Page 1 | Cost per hour for Wayfinding service.  <https://www.dementiasupport.org.uk/wayfinding> |
|  | **Day Breaks Cost**  **(2.5 hours)** | **£31.01** |  | Table 3 Daybreaks Costing  Page 2 | £74.42 for 6 hours  Cost for 2.5 hours given to compare to alternative day care costs.  <https://www.dementiasupport.org.uk/day-breaks> |
|  | **Activities** | **£13.84** |  | Table 4 Activity Costing  Page 2 | Enrichment Activities (e.g. Painting, Chair Aerobics, Knit and Natter).  <https://www.dementiasupport.org.uk/activities> |
|  | **Assisted Bathing** | **£25.27** |  | Table 5 Bathing Service Costing  Page 3 | Per contact for assisted bathing service.  <https://www.dementiasupport.org.uk/personal-care> |
|  | **Hair Dressing & Therapies (e.g., podiatry)** | **£9.46** |  | Table 6 Hair Dressing & Therapies Costing  Page 3 | Dementia friendly hair dressing service and therapies to support personal care (e.g., foot care, massage).  <https://www.dementiasupport.org.uk/personal-care> |
|  | **Cognitive Stimulation Therapy & Training**  **(group of 8)** | **£9.26** |  | Table 7 CST & Training Costs  Page 4 | CST and Training run by 3 Wayfinders. Costs for a standard group of 8 people.  <https://www.dementiasupport.org.uk/support-groups> |
|  | **Diagnostic Assessment**  **Service** | **£178.48** | **Sage House: Unit Cost Calculations**  **&**  [**Unit Costs of Health and Social Care Programme 2022-2027**](https://www.pssru.ac.uk/unitcostsreport/) | Table 8 Assessment Costs  &  Table 8.2.1 Annual and unit costs for community-based scientific and professional staff – Average band 6/7 & Table 11.3.2 Annual and unit costs for hospital-based doctors – consultant Psychiatrist | Based on a combination of Sage House floor space costing and assessment time (1 ½ hour with band 6/7 and ½ with consultant psychiatrist).  Band 6/7 average = £60.50/h  Consultant Psychiatrist = £143/h |
| **National Schedule of NHS Costs 21/22 V3:** [**https://www.england.nhs.uk/costing-in-the-nhs/national-cost-collection/**](https://www.england.nhs.uk/costing-in-the-nhs/national-cost-collection/)  **Unit Costs of Health and Social Care Programme (2022 – 2027):** [**https://www.pssru.ac.uk/unitcostsreport/**](https://www.pssru.ac.uk/unitcostsreport/) | | | | | |

**Section 5: Adherence to GRACE and CHEERS Guidelines**

**GRACE Checklist Mapping**

| GRACE Principle | Application in This Study |
| --- | --- |
| Concurrent comparator | Both groups recruited in the same time period with the same team members. |
| Adjustment for confounds | ANCOVA used to control for differences in functional independence. Extended in PLWD subsample to adjust for age, education and MoCa. |
| Outcome consistency | EQ-5D-3L and Health History protocol applied uniformly across groups. |
| Objective, standardised outcome | Standardized HRQoL measure used |
| Sensitivity Analysis | Subgroup within care partners and PLWD performed, influence of outliers checked and reported and Tweedie generalized linear model with log link and reported alongside the main analysis. |
| Follow up duration sufficient | Period was sufficient (at least 3 months) but a longer time period would have better reflected the sustained nature of the intervention. |

**CHEERS Checklist Mapping**

| **Cheers Checklist – Cost-Effectiveness Analysis of Sage House** | | | |
| --- | --- | --- | --- |
| Section / Item | Item No. | Recommendation | Reported in Manuscript (section) |
| Title | 1 | Identify the study as an economic evaluation or use more specific terms such as “cost-effectiveness analysis,” and describe the interventions compared. | Identified in title, abstract and in the aims and research question section of the introduction. |
| Abstract | 2 | Provide a structured summary of objectives, perspective, setting, methods (including study design and inputs), results (including base case and uncertainty analyses), and conclusions. | Abstract provided includes an overview of the aims, method, results and conclusions of the project. (Abstract Section) |
| Background and objectives | 3 | Provide an explicit statement of the broader context for the study. Present the study question and its relevance for health policy or practice decisions. | Introduction provides overview of the current prevalence and requirement for improved dementia care, both in terms of outcomes and costs. It also places the model within this context discussing how it can contribute to improved outcomes and the importance of understanding whether it is cost-effective. (Introduction) |
| Target population and subgroups | 4 | Describe characteristics of the base-case population and subgroups analysed, including why they were chosen. | Description of recruitment method and sample is provided in the method (Setting and Sample Subsection). |
| Setting and location | 5 | State relevant aspects of the system(s) in which the decision(s) need(s) to be made. | The project is placed within the context of the community-based setting (third sector) within the introduction and within the “Setting” section in the Methods. There is also a reflection on how this integrates with other services (e.g., NHS assessment service) within the introduction and discussion. (Introduction/ Setting and Sample Subsection/ Discussion). |
| Study perspective | 6 | Describe the perspective of the study and relate this to the costs being evaluated. | Direct costing outlined within the Costing Information Section in the Methods. Full Costing details also provided within the supplementary materials section 3. (Method, Costing Information Section and Supplementary Materials Section 3) |
| Comparators | 7 | Describe the interventions or strategies being compared and state why they were chosen. | Sage House Intervention and Usual Care Comparator described and justified in the Method Section (subsections Sage House Intervention and Usual Care Comparator) |
| Time horizon | 8 | State the time horizon(s) over which costs and consequences are being evaluated and say why appropriate. | Three-month time horizon stated and justified in Method Section (subsection Study Design). |
| Discount rate | 9 | Report the choice of discount rate(s) used for costs and outcomes and say why appropriate. | Discounting was not applied due to the short three-month time horizon. |
| Choice of health outcomes | 10 | Describe what outcomes were used as the measure(s) of benefit in the evaluation and their relevance for the type of analysis performed. | EQ-5D-3L utilities/VAS and approach to calculating QALYs outlined in Method Measures Section (Subsection Health Related Quality of Life (HRQoL)) |
| Measurement of effectiveness (single-study) | 11a | Describe fully the design features of the single effectiveness study and why it was a sufficient source of clinical effectiveness data. | Study design was outlined and justified in the Method section (subsection Study Design). |
| Measurement and valuation of preference-based outcomes | 12 | If applicable, describe the population and methods used to elicit preferences for outcomes. | Health-Related QoL (HRQoL) – UK EQ-5D-3L TTO tariff outlined in Method Section (Subsection Health Related Quality of Life (HRQoL)). |
| Estimating resources and costs | 13a | Describe approaches used to estimate resource use and valuation methods (primary or secondary). | Described in Methods section (Subsections Health Service Utilisation Questionnaire’ and ‘Costing Information’) – Also further information provided in Supplementary Information (Section 2 Health Utilisation Questionnaire, Section 3 Costing Information). |
| Currency, price date, and conversion | 14 | Report the dates of estimated resource quantities and unit costs. Describe cost-adjustment and currency-conversion methods. | Reported in Method Section - Costs valued in 2022 GBP using NHS 2021/22 schedule and PSSRU 2022 unit costs (subsection Costing Information) |
| Choice of model | 15 | Describe and give reasons for the specific type of decision-analytical model used. | Not model-based |
| Assumptions | 16 | Describe all structural or other assumptions underpinning the decision-analytical model. | Key analytical assumptions such as the timeframe (Study Design), comparability of groups (Sample Section and Discussion) and usefulness of the outcome measures (Health Related Quality of Life (HRQoL) section of Measures) have been discussed throughout. |
| Analytical methods | 17 | Describe all analytical methods supporting the evaluation (handling of data, missing data, uncertainty). | Analytical methods supporting the evaluation have been discussed in the Methods section (Subsection Data Handling and Analysis). |
| Study parameters | 18 | Report values, ranges, references, and distributions for all parameters. | Point estimates, ranges and confidence intervals have been presented for outcomes and costs (see Tables & Figures throughout). |
| Incremental costs and outcomes | 19 | Report mean costs and outcomes for each intervention, plus incremental cost-effectiveness ratios. | Mean costs and outcomes have been presented for each intervention. (Results Section). |
| Characterising uncertainty | 20 | Describe the effects of sampling uncertainty and assumptions. | Confidence intervals have been reported throughout, FDR corrected p-values reported in statistical analyses and a control analysis in the care-partner subsample was included for robustness.  Uncertainty in costs and QALYs was assessed using non-parametric bootstrapping of individual-level data. Incremental costs and QALYs were recalculated for each replication and summarised using a cost-effectiveness plane and cost-effectiveness acceptability curve (see Results Section). |
| Characterising heterogeneity | 21 | Report subgroup or variability analyses. | Sample differences described (Table 2) and differences in functional independence examined (Subsection Group Comparison of Functional Independence in the Methods Section). |
| Study findings, limitations, generalisability, and current knowledge | 22 | Summarise key findings and describe how they support the conclusions reached. Discuss limitations and generalisability. | Key findings discussed and key limitations and generalisability addressed in the Discussion Section (see Subsections Methodological Considerations and Future Directions). |
| Source of funding | 23 | Describe how the study was funded and the role of the funder in the design, conduct, and reporting of the analysis. | Included in unblinded document. |
| Conflicts of interest | 24 | Describe any potential for conflict of interest of study contributors. | Authors declare no conflicts of interest. |
